# Isopropyl 1-benzoyl-4-benzo­yloxy-2,6-di­phenyl-1,2,3,6-tetrahydropyridine-3-carboxyl­ate

**DOI:** 10.1107/S1600536814015244

**Published:** 2014-07-05

**Authors:** E. Govindan, K. Murugavel, S. Amirthaganesan, A. SubbiahPandi

**Affiliations:** a Department of Physics, Presidency College (Autonomous), Chennai 600 005, India; b Department of Chemistry, Saveetha Engineering College, Chennai, India

**Keywords:** crystal structure

## Abstract

In the title compound, C_35_H_31_NO_5_, the piperidine ring has an envelope conformation, with the phenyl-substituted C atom adjacent to the methyl­ene C atom as the flap. This flap atom deviates by 0.633 (2) Å from the mean plane of the other five essentially coplanar atoms in the ring (r.m.s. deviation = 0.044 Å). Intra­molecular C—H⋯O hydrogen bonds form *S*(7) and *S*(9) ring motifs. In the crystal, mol­ecules are linked by pairs of C—H⋯O hydrogen bonds, forming inversion dimers with *R* ^2^_2_(16) loops.

## Related literature   

For general background to piperidine derivatives, see: Mishra & Ghosh (2011 ▶); Ramachandran *et al.* (2011 ▶); Natarajan & Mathews (2011 ▶). For hydrogen-bond motifs, see: Bernstein *et al.* (1995 ▶).
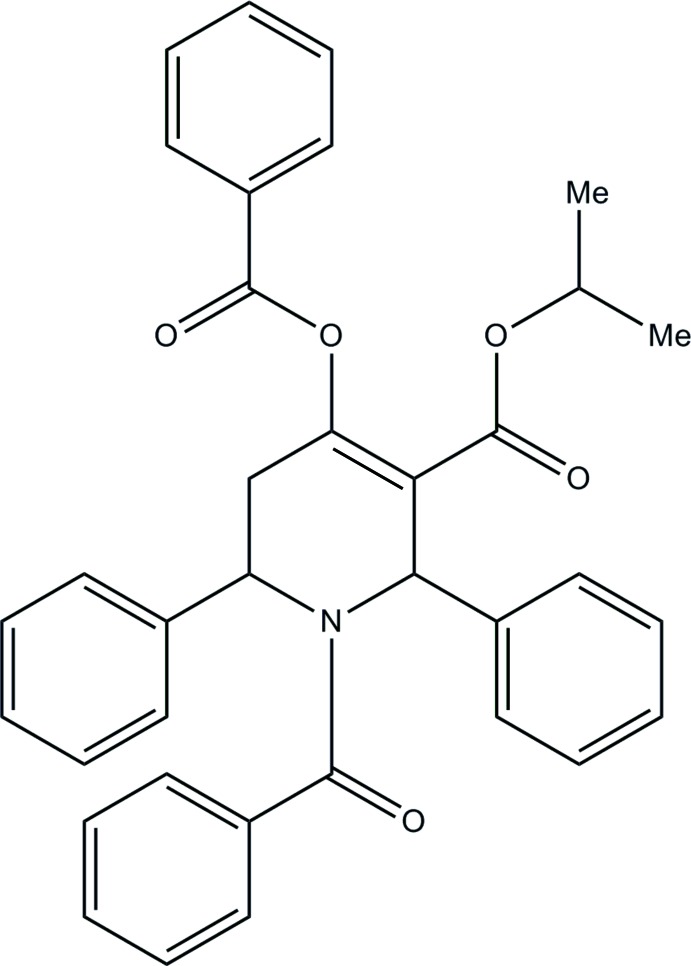


## Experimental   

### 

#### Crystal data  

C_35_H_31_NO_5_
*M* *_r_* = 545.61
Triclinic, 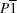

*a* = 10.1788 (6) Å
*b* = 11.4325 (7) Å
*c* = 13.1395 (9) Å
α = 81.847 (2)°
β = 86.662 (3)°
γ = 69.654 (2)°
*V* = 1419.08 (15) Å^3^
*Z* = 2
Mo *K*α radiation
μ = 0.09 mm^−1^
*T* = 293 K
0.22 × 0.19 × 0.17 mm

#### Data collection  

Bruker SMART APEXII CCD diffractometer
Absorption correction: multi-scan (*SADABS*; Bruker, 2008 ▶) *T*_min_ = 0.952, *T*_max_ = 0.959
25561 measured reflections
4996 independent reflections
3876 reflections with *I* > 2σ(*I*)
*R*_int_ = 0.028

#### Refinement  

*R*[*F* ^2^ > 2σ(*F* ^2^)] = 0.035
*wR*(*F* ^2^) = 0.109
*S* = 1.02
4996 reflections
372 parameters
H-atom parameters constrained
Δρ_max_ = 0.21 e Å^−3^
Δρ_min_ = −0.17 e Å^−3^

### 

Data collection: *APEX2* (Bruker, 2008 ▶); cell refinement: *SAINT* (Bruker, 2008 ▶); data reduction: *SAINT*; program(s) used to solve structure: *SHELXS97* (Sheldrick, 2008 ▶); program(s) used to refine structure: *SHELXL97* (Sheldrick, 2008 ▶); molecular graphics: *ORTEP-3 for Windows* (Farrugia, 2012 ▶) and *Mercury* (Macrae *et al.*, 2008 ▶); software used to prepare material for publication: *SHELXL97* and *PLATON* (Spek, 2009 ▶).

## Supplementary Material

Crystal structure: contains datablock(s) global, I. DOI: 10.1107/S1600536814015244/nk2218sup1.cif

Structure factors: contains datablock(s) I. DOI: 10.1107/S1600536814015244/nk2218Isup2.hkl

Supporting information file. DOI: 10.1107/S1600536814015244/nk2218Isup3.cml

CCDC reference: 1010935

Additional supporting information:  crystallographic information; 3D view; checkCIF report

## Figures and Tables

**Table 1:** Hydrogen-bond geometry (Å, °)

*D*—H⋯*A*	*D*—H	H⋯*A*	*D*⋯*A*	*D*—H⋯*A*
C10—H10⋯O5^i^	0.93	2.57	3.500 (2)	173
C11—H11⋯O5	0.93	2.53	3.171 (2)	126
C24—H24⋯O4	0.93	2.36	3.2898 (19)	179

Symmetry code: (i) 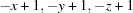.

**Fig. 1.**
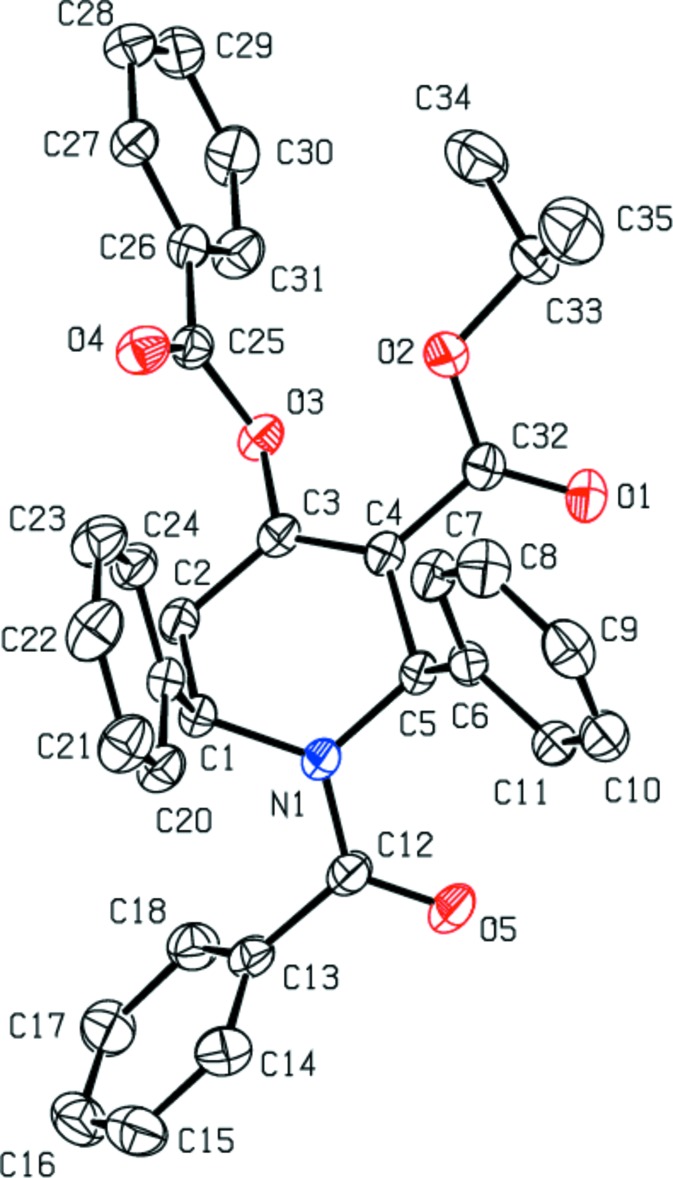
The molecular structure of the title compound, showing the atomic numbering and displacement ellipsoids drawn at 30% probability level.

**Fig. 2.**
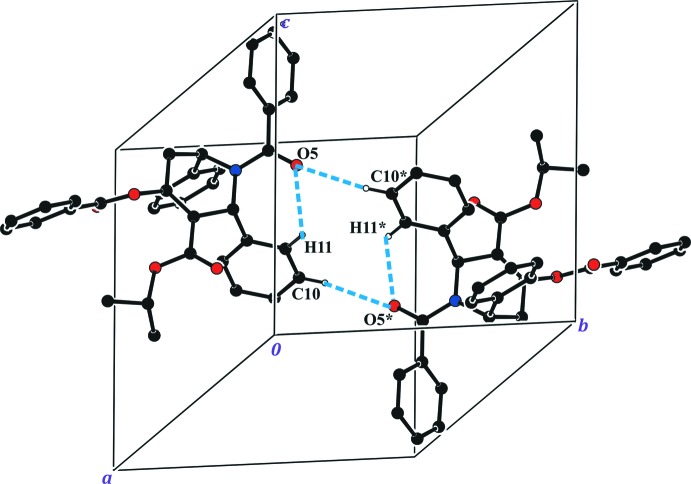
The crystal structure showing the centrosymmetric hydrogen bond motif R22(16). For the sake of clarity, the H atoms not involved in the motif have been omitted.

## supplementary crystallographic information

### S1. Comment

Piperidine iminocyclitols have shown significant pharmacological results against HIV and in cancer therapy. Alkaloids containing the piperidine nucleus exhibited a promising wide range of biological activities such as antimicrobial, antiparasitic, cytotoxicity, anti-inflammatory, pesticidal and anti-HIV-1 properties (Mishra and Ghosh, 2011). The motivation for the biological trial arises as piperidine derivatives are an important class of heterocyclic compounds with potent pharmacological/biological activities (Ramachandran *et al.*, 2011). Against this background, and in order to obtain detailed information on molecular conformations in the solid state, an X-ray study of the title compound was carried out.

X-Ray analysis confirms the molecular structure and atom connectivity as illustrated in Fig. 1. The bond lengths N1—C1=1.477 (2), O5—C12=1.221 (2) are normal and comparable with the corresponding values observed in the related structure of Ethyl 3-methyl-2,6-diphenylpiperidine-1-carboxylate (Natarajan & Mathews, 2011). The sum of the bond angle around atom N1 (359.9°) of the piperidine ring indicates *sp*^2^ hybridization. Both phenyl rings are axial substituents on the piperidine ring and the angle between least-squares mean planes fitted through both rings is 45.28 (8)°.

The acetate group is almost extended conformation which can be seen from the torsion angle [C4—C32—O2—C33 = -179.6 (2)°] The piperidine ring adopts a distorted envelope conformation. In the crystal, molecules are linked into cyclic centrosymmetric dimers *via* C10–H10···O5 hydrogen bonds with the ring motif *R*^2^_2_(16), which propagates along the *b* axis, as shown in Fig. 2. An intramolecular C11—H11···O5 hydrogen bond form an S(7) [H11,C11,C6,C5,N1,C12 and O5] ring motif (Bernstein *et al.*, 1995).

### S2. Experimental

3-Isopropyl-2,6-diphenylpiperidin-4-one carboxylate were syntheized by Mannich condensation of benzaldehyde with isopropyl acetoacetate. Isopropyl acetoacetate (0.01 mol), benzaldehydes (0.02 mol) and ammonium acetate (0.01 mol) were taken in a 500 ml round bottom flask. Further ethanol (25 ml) was added to the flask, warmed for about 10 min. and set aside until the product crystallized. The product obtained was filtered and the solid product was collected and washed with cold water. The product was dried at room temperature and recrystallized from ethanol to obtain 3-isopropyl-2,6-diphenylpiperidin-4-one carboxylate. Equimolar amounts of 3-isopropyl-2,6-diphenylpiperidin -4-one carboxylate (3.37 g, 100 mmol), benzoyl chloride (2 ml, 100 mmol) ethylene triamine (2 ml, 200 mmol) and benzene (50 ml) were heated to reflux for 6–10 h. The reaction was monitored by TLC, after completion of the reaction solvent was washed with 2 N HCl followed by water. solvent was removed under vacuum and the residue was recrystalized form ethanol. The yield of the isolated product was 2.65 g (70%).

### S3. Refinement

All H atoms were fixed geometrically and allowed to ride on their parent C atoms, with C—H distances fixed in the range 0.93–0.97 Å with *U*_iso_(H) = 1.5*U*_eq_(C) for methyl H atoms and 1.2*U*_eq_(C) for all other H atoms.

### Crystal data

**Table:**

C_35_H_31_NO_5_	*V* = 1419.08 (15) Å^3^
*M**_r_* = 545.61	*Z* = 2
Triclinic, *P*1	*F*(000) = 576
Hall symbol: -P 1	*D*_x_ = 1.277 Mg m^−^^3^
*a* = 10.1788 (6) Å	Mo *K*α radiation, λ = 0.71073 Å
*b* = 11.4325 (7) Å	µ = 0.09 mm^−^^1^
*c* = 13.1395 (9) Å	*T* = 293 K
α = 81.847 (2)°	Block, white
β = 86.662 (3)°	0.22 × 0.19 × 0.17 mm
γ = 69.654 (2)°	

### Data collection

**Table:**

Bruker SMART APEXII CCD diffractometer	4996 independent reflections
Radiation source: fine-focus sealed tube	3876 reflections with *I* > 2σ(*I*)
Graphite monochromator	*R*_int_ = 0.028
ω and φ scans	θ_max_ = 25.0°, θ_min_ = 2.1°
Absorption correction: multi-scan (*SADABS*; Bruker, 2008)	*h* = −11→12
*T*_min_ = 0.952, *T*_max_ = 0.959	*k* = −13→13
25561 measured reflections	*l* = −15→15

### Refinement

**Table:**

Refinement on *F*^2^	Primary atom site location: structure-invariant direct methods
Least-squares matrix: full	Secondary atom site location: difference Fourier map
*R*[*F*^2^ > 2σ(*F*^2^)] = 0.035	Hydrogen site location: inferred from neighbouring sites
*wR*(*F*^2^) = 0.109	H-atom parameters constrained
*S* = 1.02	*w* = 1/[σ^2^(*F*_o_^2^) + (0.0614*P*)^2^ + 0.1791*P*] where *P* = (*F*_o_^2^ + 2*F*_c_^2^)/3
4996 reflections	(Δ/σ)_max_ = 0.001
372 parameters	Δρ_max_ = 0.21 e Å^−^^3^
0 restraints	Δρ_min_ = −0.17 e Å^−^^3^

### Special details

**Table:**

Geometry. All e.s.d.'s (except the e.s.d. in the dihedral angle between two l.s. planes) are estimated using the full covariance matrix. The cell e.s.d.'s are taken into account individually in the estimation of e.s.d.'s in distances, angles and torsion angles; correlations between e.s.d.'s in cell parameters are only used when they are defined by crystal symmetry. An approximate (isotropic) treatment of cell e.s.d.'s is used for estimating e.s.d.'s involving l.s. planes.
Refinement. Refinement of *F*^2^ against ALL reflections. The weighted *R*-factor *wR* and goodness of fit *S* are based on *F*^2^, conventional *R*-factors *R* are based on *F*, with *F* set to zero for negative *F*^2^. The threshold expression of *F*^2^ > σ(*F*^2^) is used only for calculating *R*-factors(gt) *etc*. and is not relevant to the choice of reflections for refinement. *R*-factors based on *F*^2^ are statistically about twice as large as those based on *F*, and *R*- factors based on ALL data will be even larger.

### Fractional atomic coordinates and isotropic or equivalent isotropic displacement parameters (Å^2^)

**Table:**

	*x*	*y*	*z*	*U*_iso_*/*U*_eq_	
C20	0.96506 (16)	0.08716 (14)	0.67253 (11)	0.0519 (4)	
H20	0.8967	0.0763	0.6347	0.062*	
C21	1.09259 (18)	0.08065 (17)	0.62695 (13)	0.0646 (5)	
H21	1.1096	0.0652	0.5589	0.078*	
C22	1.19444 (18)	0.09692 (18)	0.68161 (14)	0.0673 (5)	
H22	1.2805	0.0924	0.6510	0.081*	
C23	1.16822 (16)	0.11983 (17)	0.78179 (13)	0.0614 (4)	
H23	1.2365	0.1317	0.8189	0.074*	
C24	1.04077 (15)	0.12537 (14)	0.82808 (12)	0.0490 (4)	
H24	1.0246	0.1399	0.8963	0.059*	
C19	0.93736 (14)	0.10950 (12)	0.77359 (10)	0.0405 (3)	
C1	0.79798 (13)	0.10671 (12)	0.82094 (10)	0.0395 (3)	
H1	0.7885	0.0281	0.8069	0.047*	
C2	0.78325 (14)	0.10737 (12)	0.93686 (10)	0.0415 (3)	
H2B	0.7113	0.0729	0.9625	0.050*	
H2A	0.8709	0.0539	0.9692	0.050*	
C3	0.74623 (13)	0.23572 (12)	0.96537 (10)	0.0386 (3)	
C4	0.69662 (13)	0.34103 (12)	0.90037 (10)	0.0386 (3)	
C5	0.65643 (14)	0.33967 (12)	0.79121 (10)	0.0392 (3)	
H5	0.5551	0.3845	0.7874	0.047*	
C6	0.72113 (14)	0.41264 (12)	0.70991 (10)	0.0407 (3)	
C7	0.84932 (16)	0.42433 (14)	0.72253 (12)	0.0520 (4)	
H7	0.9004	0.3847	0.7818	0.062*	
C8	0.90280 (18)	0.49434 (16)	0.64800 (13)	0.0622 (4)	
H8	0.9897	0.5011	0.6571	0.075*	
C9	0.8275 (2)	0.55393 (15)	0.56055 (13)	0.0617 (4)	
H9	0.8636	0.6008	0.5104	0.074*	
C10	0.6998 (2)	0.54443 (14)	0.54719 (11)	0.0586 (4)	
H10	0.6488	0.5851	0.4881	0.070*	
C11	0.64622 (16)	0.47421 (13)	0.62156 (11)	0.0482 (4)	
H11	0.5590	0.4683	0.6121	0.058*	
C32	0.66009 (14)	0.46966 (13)	0.93018 (10)	0.0428 (3)	
C33	0.72277 (18)	0.59541 (14)	1.03681 (13)	0.0607 (4)	
H33	0.6229	0.6459	1.0317	0.073*	
C34	0.7649 (3)	0.56499 (19)	1.14784 (15)	0.0892 (6)	
H34A	0.8630	0.5163	1.1528	0.134*	
H34B	0.7118	0.5175	1.1849	0.134*	
H34C	0.7469	0.6417	1.1768	0.134*	
C35	0.8054 (3)	0.6625 (2)	0.97090 (19)	0.0940 (7)	
H35A	0.7820	0.7460	0.9887	0.141*	
H35B	0.7838	0.6671	0.9000	0.141*	
H35C	0.9036	0.6175	0.9816	0.141*	
N1	0.67724 (11)	0.21014 (10)	0.77234 (8)	0.0401 (3)	
O1	0.56508 (11)	0.55833 (9)	0.89218 (8)	0.0572 (3)	
O2	0.74703 (10)	0.47477 (9)	1.00008 (8)	0.0530 (3)	
O3	0.74902 (9)	0.24164 (9)	1.07066 (7)	0.0441 (2)	
O4	0.98397 (11)	0.18061 (10)	1.06863 (8)	0.0535 (3)	
O5	0.48623 (13)	0.27894 (10)	0.67147 (10)	0.0748 (4)	
C25	0.87486 (15)	0.21460 (12)	1.11539 (11)	0.0409 (3)	
C26	0.86017 (15)	0.23327 (12)	1.22445 (10)	0.0430 (3)	
C13	0.58887 (14)	0.05921 (14)	0.70980 (11)	0.0468 (4)	
C12	0.58068 (15)	0.19148 (14)	0.71550 (11)	0.0484 (4)	
C31	0.73276 (17)	0.26105 (15)	1.27588 (12)	0.0569 (4)	
H31	0.6523	0.2683	1.2417	0.068*	
C27	0.97936 (17)	0.22454 (14)	1.27551 (12)	0.0523 (4)	
H27	1.0651	0.2061	1.2410	0.063*	
C14	0.62173 (17)	0.00964 (16)	0.61776 (13)	0.0605 (4)	
H14	0.6427	0.0573	0.5599	0.073*	
C18	0.55352 (17)	−0.01192 (15)	0.79366 (13)	0.0552 (4)	
H18	0.5299	0.0206	0.8558	0.066*	
C17	0.55295 (18)	−0.13089 (16)	0.78597 (15)	0.0656 (5)	
H17	0.5275	−0.1776	0.8425	0.079*	
C30	0.7256 (2)	0.27799 (17)	1.37821 (13)	0.0691 (5)	
H30	0.6404	0.2951	1.4134	0.083*	
C28	0.9705 (2)	0.24314 (16)	1.37712 (13)	0.0638 (5)	
H28	1.0503	0.2377	1.4113	0.077*	
C29	0.8442 (2)	0.26963 (16)	1.42834 (13)	0.0690 (5)	
H29	0.8388	0.2820	1.4971	0.083*	
C16	0.58985 (19)	−0.18005 (17)	0.69509 (16)	0.0696 (5)	
H16	0.5920	−0.2611	0.6904	0.084*	
C15	0.62343 (19)	−0.11043 (18)	0.61150 (16)	0.0704 (5)	
H15	0.6477	−0.1440	0.5498	0.084*	

### Atomic displacement parameters (Å^2^)

**Table:**

	*U*^11^	*U*^22^	*U*^33^	*U*^12^	*U*^13^	*U*^23^
C20	0.0513 (9)	0.0568 (9)	0.0435 (9)	−0.0144 (7)	−0.0085 (7)	−0.0009 (7)
C21	0.0614 (11)	0.0797 (12)	0.0432 (9)	−0.0157 (9)	0.0018 (8)	−0.0006 (8)
C22	0.0450 (9)	0.0867 (13)	0.0607 (11)	−0.0155 (9)	0.0049 (8)	0.0003 (9)
C23	0.0402 (9)	0.0773 (11)	0.0652 (11)	−0.0170 (8)	−0.0040 (8)	−0.0111 (9)
C24	0.0401 (8)	0.0544 (9)	0.0483 (9)	−0.0094 (7)	−0.0061 (7)	−0.0082 (7)
C19	0.0381 (7)	0.0337 (7)	0.0433 (8)	−0.0055 (6)	−0.0063 (6)	−0.0001 (6)
C1	0.0383 (7)	0.0337 (7)	0.0432 (8)	−0.0086 (6)	−0.0095 (6)	−0.0002 (5)
C2	0.0384 (7)	0.0381 (7)	0.0443 (8)	−0.0107 (6)	−0.0072 (6)	0.0032 (6)
C3	0.0324 (7)	0.0455 (7)	0.0350 (7)	−0.0104 (6)	−0.0019 (5)	−0.0027 (6)
C4	0.0333 (7)	0.0388 (7)	0.0404 (7)	−0.0090 (6)	−0.0033 (6)	−0.0021 (6)
C5	0.0349 (7)	0.0350 (7)	0.0430 (8)	−0.0066 (5)	−0.0080 (6)	−0.0003 (5)
C6	0.0444 (8)	0.0334 (7)	0.0402 (8)	−0.0081 (6)	−0.0023 (6)	−0.0043 (5)
C7	0.0480 (9)	0.0497 (8)	0.0536 (9)	−0.0139 (7)	−0.0062 (7)	0.0039 (7)
C8	0.0566 (10)	0.0623 (10)	0.0680 (11)	−0.0247 (8)	0.0062 (8)	−0.0013 (8)
C9	0.0815 (12)	0.0510 (9)	0.0515 (10)	−0.0248 (9)	0.0148 (9)	−0.0041 (7)
C10	0.0828 (12)	0.0497 (9)	0.0387 (8)	−0.0182 (8)	−0.0062 (8)	−0.0005 (7)
C11	0.0579 (9)	0.0428 (8)	0.0416 (8)	−0.0138 (7)	−0.0097 (7)	−0.0030 (6)
C32	0.0387 (8)	0.0426 (8)	0.0432 (8)	−0.0103 (6)	0.0004 (6)	−0.0031 (6)
C33	0.0617 (10)	0.0438 (8)	0.0788 (12)	−0.0160 (7)	−0.0105 (9)	−0.0162 (8)
C34	0.1236 (18)	0.0702 (12)	0.0775 (14)	−0.0304 (12)	−0.0076 (13)	−0.0259 (10)
C35	0.1025 (16)	0.0748 (13)	0.1143 (18)	−0.0469 (12)	−0.0037 (13)	−0.0003 (12)
N1	0.0372 (6)	0.0365 (6)	0.0442 (7)	−0.0098 (5)	−0.0112 (5)	−0.0007 (5)
O1	0.0547 (6)	0.0426 (6)	0.0625 (7)	−0.0012 (5)	−0.0115 (5)	−0.0040 (5)
O2	0.0485 (6)	0.0433 (5)	0.0659 (7)	−0.0095 (5)	−0.0121 (5)	−0.0138 (5)
O3	0.0385 (5)	0.0524 (6)	0.0362 (5)	−0.0103 (4)	−0.0044 (4)	−0.0017 (4)
O4	0.0424 (6)	0.0679 (7)	0.0501 (6)	−0.0175 (5)	0.0011 (5)	−0.0118 (5)
O5	0.0707 (8)	0.0547 (7)	0.0959 (9)	−0.0160 (6)	−0.0504 (7)	0.0059 (6)
C25	0.0406 (8)	0.0381 (7)	0.0432 (8)	−0.0138 (6)	−0.0051 (6)	0.0002 (6)
C26	0.0478 (8)	0.0384 (7)	0.0410 (8)	−0.0131 (6)	−0.0058 (6)	−0.0011 (6)
C13	0.0383 (8)	0.0504 (8)	0.0533 (9)	−0.0164 (6)	−0.0131 (6)	−0.0038 (7)
C12	0.0448 (8)	0.0490 (8)	0.0505 (9)	−0.0156 (7)	−0.0148 (7)	0.0013 (7)
C31	0.0519 (9)	0.0659 (10)	0.0482 (9)	−0.0143 (8)	−0.0031 (7)	−0.0063 (7)
C27	0.0542 (9)	0.0510 (8)	0.0509 (9)	−0.0172 (7)	−0.0117 (7)	−0.0016 (7)
C14	0.0596 (10)	0.0691 (11)	0.0584 (10)	−0.0285 (8)	−0.0030 (8)	−0.0091 (8)
C18	0.0555 (9)	0.0579 (9)	0.0561 (10)	−0.0241 (8)	−0.0056 (7)	−0.0064 (7)
C17	0.0631 (11)	0.0602 (10)	0.0787 (12)	−0.0308 (8)	−0.0044 (9)	0.0007 (9)
C30	0.0712 (12)	0.0734 (12)	0.0476 (10)	−0.0059 (9)	0.0054 (9)	−0.0097 (8)
C28	0.0780 (13)	0.0586 (10)	0.0557 (10)	−0.0214 (9)	−0.0236 (9)	−0.0063 (8)
C29	0.0971 (15)	0.0557 (10)	0.0447 (9)	−0.0107 (9)	−0.0136 (10)	−0.0104 (8)
C16	0.0620 (11)	0.0574 (10)	0.0978 (15)	−0.0262 (9)	−0.0010 (10)	−0.0222 (10)
C15	0.0672 (11)	0.0764 (12)	0.0767 (12)	−0.0284 (10)	0.0042 (9)	−0.0319 (10)

### Geometric parameters (Å, º)

**Table:**

C20—C21	1.380 (2)	C33—O2	1.4609 (17)
C20—C19	1.382 (2)	C33—C35	1.490 (3)
C20—H20	0.9300	C33—C34	1.503 (2)
C21—C22	1.373 (2)	C33—H33	0.9800
C21—H21	0.9300	C34—H34A	0.9600
C22—C23	1.371 (2)	C34—H34B	0.9600
C22—H22	0.9300	C34—H34C	0.9600
C23—C24	1.385 (2)	C35—H35A	0.9600
C23—H23	0.9300	C35—H35B	0.9600
C24—C19	1.3823 (19)	C35—H35C	0.9600
C24—H24	0.9300	N1—C12	1.3586 (17)
C19—C1	1.5244 (19)	O3—C25	1.3574 (16)
C1—N1	1.4775 (16)	O4—C25	1.2042 (16)
C1—C2	1.5225 (19)	O5—C12	1.2210 (17)
C1—H1	0.9800	C25—C26	1.4700 (19)
C2—C3	1.4800 (18)	C26—C31	1.383 (2)
C2—H2B	0.9700	C26—C27	1.387 (2)
C2—H2A	0.9700	C13—C18	1.381 (2)
C3—C4	1.3303 (18)	C13—C14	1.382 (2)
C3—O3	1.3970 (16)	C13—C12	1.498 (2)
C4—C32	1.4901 (19)	C31—C30	1.380 (2)
C4—C5	1.5179 (19)	C31—H31	0.9300
C5—N1	1.4761 (16)	C27—C28	1.374 (2)
C5—C6	1.5225 (19)	C27—H27	0.9300
C5—H5	0.9800	C14—C15	1.381 (2)
C6—C7	1.379 (2)	C14—H14	0.9300
C6—C11	1.3866 (19)	C18—C17	1.380 (2)
C7—C8	1.384 (2)	C18—H18	0.9300
C7—H7	0.9300	C17—C16	1.369 (3)
C8—C9	1.375 (2)	C17—H17	0.9300
C8—H8	0.9300	C30—C29	1.377 (3)
C9—C10	1.364 (2)	C30—H30	0.9300
C9—H9	0.9300	C28—C29	1.372 (3)
C10—C11	1.385 (2)	C28—H28	0.9300
C10—H10	0.9300	C29—H29	0.9300
C11—H11	0.9300	C16—C15	1.362 (3)
C32—O1	1.2013 (16)	C16—H16	0.9300
C32—O2	1.3326 (17)	C15—H15	0.9300
			
C21—C20—C19	121.03 (15)	O2—C33—C34	106.04 (13)
C21—C20—H20	119.5	C35—C33—C34	113.85 (17)
C19—C20—H20	119.5	O2—C33—H33	109.5
C22—C21—C20	120.23 (16)	C35—C33—H33	109.5
C22—C21—H21	119.9	C34—C33—H33	109.5
C20—C21—H21	119.9	C33—C34—H34A	109.5
C23—C22—C21	119.41 (16)	C33—C34—H34B	109.5
C23—C22—H22	120.3	H34A—C34—H34B	109.5
C21—C22—H22	120.3	C33—C34—H34C	109.5
C22—C23—C24	120.53 (16)	H34A—C34—H34C	109.5
C22—C23—H23	119.7	H34B—C34—H34C	109.5
C24—C23—H23	119.7	C33—C35—H35A	109.5
C19—C24—C23	120.50 (14)	C33—C35—H35B	109.5
C19—C24—H24	119.8	H35A—C35—H35B	109.5
C23—C24—H24	119.8	C33—C35—H35C	109.5
C20—C19—C24	118.30 (13)	H35A—C35—H35C	109.5
C20—C19—C1	118.08 (12)	H35B—C35—H35C	109.5
C24—C19—C1	123.50 (13)	C12—N1—C5	118.36 (10)
N1—C1—C2	107.94 (10)	C12—N1—C1	123.35 (11)
N1—C1—C19	112.29 (10)	C5—N1—C1	118.21 (10)
C2—C1—C19	116.08 (11)	C32—O2—C33	118.20 (11)
N1—C1—H1	106.7	C25—O3—C3	118.50 (10)
C2—C1—H1	106.7	O4—C25—O3	122.40 (13)
C19—C1—H1	106.7	O4—C25—C26	125.44 (13)
C3—C2—C1	111.93 (10)	O3—C25—C26	112.16 (12)
C3—C2—H2B	109.2	C31—C26—C27	119.87 (14)
C1—C2—H2B	109.2	C31—C26—C25	122.37 (13)
C3—C2—H2A	109.2	C27—C26—C25	117.75 (13)
C1—C2—H2A	109.2	C18—C13—C14	118.73 (14)
H2B—C2—H2A	107.9	C18—C13—C12	120.89 (14)
C4—C3—O3	119.97 (12)	C14—C13—C12	120.19 (14)
C4—C3—C2	124.51 (12)	O5—C12—N1	122.03 (13)
O3—C3—C2	115.02 (10)	O5—C12—C13	119.46 (12)
C3—C4—C32	123.90 (12)	N1—C12—C13	118.48 (12)
C3—C4—C5	121.79 (12)	C30—C31—C26	119.66 (16)
C32—C4—C5	113.95 (11)	C30—C31—H31	120.2
N1—C5—C4	110.68 (10)	C26—C31—H31	120.2
N1—C5—C6	114.62 (11)	C28—C27—C26	119.90 (16)
C4—C5—C6	113.74 (11)	C28—C27—H27	120.1
N1—C5—H5	105.6	C26—C27—H27	120.1
C4—C5—H5	105.6	C15—C14—C13	120.17 (16)
C6—C5—H5	105.6	C15—C14—H14	119.9
C7—C6—C11	118.44 (13)	C13—C14—H14	119.9
C7—C6—C5	122.49 (12)	C17—C18—C13	120.56 (16)
C11—C6—C5	119.02 (12)	C17—C18—H18	119.7
C6—C7—C8	120.72 (14)	C13—C18—H18	119.7
C6—C7—H7	119.6	C16—C17—C18	119.94 (17)
C8—C7—H7	119.6	C16—C17—H17	120.0
C9—C8—C7	120.01 (16)	C18—C17—H17	120.0
C9—C8—H8	120.0	C29—C30—C31	120.16 (17)
C7—C8—H8	120.0	C29—C30—H30	119.9
C10—C9—C8	120.09 (15)	C31—C30—H30	119.9
C10—C9—H9	120.0	C29—C28—C27	120.19 (16)
C8—C9—H9	120.0	C29—C28—H28	119.9
C9—C10—C11	120.01 (15)	C27—C28—H28	119.9
C9—C10—H10	120.0	C28—C29—C30	120.21 (16)
C11—C10—H10	120.0	C28—C29—H29	119.9
C10—C11—C6	120.73 (15)	C30—C29—H29	119.9
C10—C11—H11	119.6	C15—C16—C17	120.10 (16)
C6—C11—H11	119.6	C15—C16—H16	120.0
O1—C32—O2	124.75 (13)	C17—C16—H16	120.0
O1—C32—C4	122.82 (13)	C16—C15—C14	120.44 (17)
O2—C32—C4	112.39 (11)	C16—C15—H15	119.8
O2—C33—C35	108.43 (15)	C14—C15—H15	119.8
			
C19—C20—C21—C22	−0.3 (2)	C4—C5—N1—C1	−36.21 (15)
C20—C21—C22—C23	−0.1 (3)	C6—C5—N1—C1	94.06 (13)
C21—C22—C23—C24	0.6 (3)	C2—C1—N1—C12	−117.56 (14)
C22—C23—C24—C19	−0.8 (2)	C19—C1—N1—C12	113.23 (14)
C21—C20—C19—C24	0.1 (2)	C2—C1—N1—C5	59.05 (14)
C21—C20—C19—C1	−175.99 (13)	C19—C1—N1—C5	−70.17 (14)
C23—C24—C19—C20	0.5 (2)	O1—C32—O2—C33	2.6 (2)
C23—C24—C19—C1	176.31 (14)	C4—C32—O2—C33	−179.61 (12)
C20—C19—C1—N1	−66.15 (15)	C35—C33—O2—C32	−91.49 (17)
C24—C19—C1—N1	118.01 (14)	C34—C33—O2—C32	145.87 (15)
C20—C19—C1—C2	169.00 (12)	C4—C3—O3—C25	−108.81 (14)
C24—C19—C1—C2	−6.84 (18)	C2—C3—O3—C25	78.95 (14)
N1—C1—C2—C3	−45.79 (14)	C3—O3—C25—O4	−3.28 (18)
C19—C1—C2—C3	81.25 (14)	C3—O3—C25—C26	176.06 (10)
C1—C2—C3—C4	16.21 (18)	O4—C25—C26—C31	−173.77 (14)
C1—C2—C3—O3	−171.96 (10)	O3—C25—C26—C31	6.91 (18)
O3—C3—C4—C32	8.7 (2)	O4—C25—C26—C27	7.4 (2)
C2—C3—C4—C32	−179.86 (12)	O3—C25—C26—C27	−171.88 (11)
O3—C3—C4—C5	−163.98 (11)	C5—N1—C12—O5	7.5 (2)
C2—C3—C4—C5	7.5 (2)	C1—N1—C12—O5	−175.88 (14)
C3—C4—C5—N1	1.40 (17)	C5—N1—C12—C13	−170.63 (12)
C32—C4—C5—N1	−171.94 (10)	C1—N1—C12—C13	6.0 (2)
C3—C4—C5—C6	−129.34 (13)	C18—C13—C12—O5	−106.54 (18)
C32—C4—C5—C6	57.32 (15)	C14—C13—C12—O5	68.5 (2)
N1—C5—C6—C7	−98.31 (15)	C18—C13—C12—N1	71.66 (19)
C4—C5—C6—C7	30.45 (18)	C14—C13—C12—N1	−113.33 (16)
N1—C5—C6—C11	84.44 (15)	C27—C26—C31—C30	−1.0 (2)
C4—C5—C6—C11	−146.80 (13)	C25—C26—C31—C30	−179.75 (14)
C11—C6—C7—C8	−1.0 (2)	C31—C26—C27—C28	0.2 (2)
C5—C6—C7—C8	−178.23 (14)	C25—C26—C27—C28	179.03 (13)
C6—C7—C8—C9	0.5 (2)	C18—C13—C14—C15	−2.1 (2)
C7—C8—C9—C10	0.2 (3)	C12—C13—C14—C15	−177.24 (14)
C8—C9—C10—C11	−0.3 (2)	C14—C13—C18—C17	0.9 (2)
C9—C10—C11—C6	−0.2 (2)	C12—C13—C18—C17	175.96 (14)
C7—C6—C11—C10	0.8 (2)	C13—C18—C17—C16	1.1 (3)
C5—C6—C11—C10	178.19 (13)	C26—C31—C30—C29	1.2 (3)
C3—C4—C32—O1	−146.00 (15)	C26—C27—C28—C29	0.4 (2)
C5—C4—C32—O1	27.18 (18)	C27—C28—C29—C30	−0.1 (3)
C3—C4—C32—O2	36.14 (18)	C31—C30—C29—C28	−0.7 (3)
C5—C4—C32—O2	−150.68 (12)	C18—C17—C16—C15	−1.8 (3)
C4—C5—N1—C12	140.57 (12)	C17—C16—C15—C14	0.5 (3)
C6—C5—N1—C12	−89.16 (14)	C13—C14—C15—C16	1.4 (3)

### Hydrogen-bond geometry (Å, º)

**Table:**

*D*—H···*A*	*D*—H	H···*A*	*D*···*A*	*D*—H···*A*
C10—H10···O5^i^	0.93	2.57	3.500 (2)	173
C11—H11···O5	0.93	2.53	3.171 (2)	126
C24—H24···O4	0.93	2.36	3.2898 (19)	179

Symmetry code: (i) −*x*+1, −*y*+1, −*z*+1.
